# A retrospective study of *Uncinaria stenocephala* in domestic dogs: Age, sex distribution, and risk factors

**DOI:** 10.14202/vetworld.2021.265-269

**Published:** 2021-01-29

**Authors:** Michail Yur’iyevich Shchelkanov, Tatyana Vladimirovna Tabakaeva, Pavel Vasilevich Fomenko, Ekaterina Michailovna Kim, Anton Vadimovich Tabakaev, Irina Vyacheslavovna Galkina

**Affiliations:** 1 Far Eastern Federal University, School of Biomedicine, Vladivostok, Russia; 2 Federal Scientific Center of Terrestrial Biodiversity of Eastern Asia, Far Eastern Branch of Russian Academy of Sciences, Stoletiya Vladivostoku, 159/1, Vladivostok, Primorsky Krai, Russia; 3 National Scientific Center of Marine Biology, Far Eastern Branch of Russian Academy of Sciences, Palchevskogo, 17, Vladivostok, Primorsky Krai, Russia; 4 Amur Branch of Russian Department of World Wide Fund, Verkhneportovaya, 18A, Vladivostok, Primorsky Krai, Russia

**Keywords:** canine hookworm, dogs, infection, *Uncinaria stenocephala*

## Abstract

**Background and Aim:**

*Uncinaria* infection often appears in domestic dogs. In the present study, parasitological examination of fecal samples from 782 dogs were analyzed for the presence of *Uncinaria stenocephala*.

**Materials and Methods:**

Fecal samples were analyzed by means of a standardized flotation method using a saturated salt solution containing NaNO_3_ (specific gravity 1.38), with a centrifugation step.

**Results:**

The highest prevalence rates were found among young adult dogs (8.3%), followed by puppies (5.4%); the lowest prevalence rates were found in dogs older than 3 years (4.3%). The prevalence was 5.8% among female dogs and 7.2% in male dogs. Coinfections with roundworms and protozoan parasites were frequently observed in *U. stenocephala*-positive dogs (15%). In total, three types of coinfections were registered. Coinfection of *U. stenocephala* + *Sarcocystids oocysts* was recorded in 19.1% of the dogs (n=10). This may relate to higher prevalence of *S. oocysts* in dogs (n=153; 19.5%). There were two cases of coinfection of *U. stenocephala* + *Toxocara canis* (3.9%), which may relate to low prevalence of *T. canis* (3.9 %). One case of coinfection of *Dipylidium caninum* *+* *U. stenocephala* (0.1%) also appeared.

**Conclusion:**

The present study showed that male dogs and young dogs were most susceptible to *U. stenocephala* infection.

## Introduction

Hookworms are the most prevalent intestinal parasites of domestic dogs and cats. The most common canine hookworms (*Nematoda* and Strongylidae) are *Ancylostoma caninum*, *Ancylostoma braziliense*, *Ancylostoma ceylanicum*, and *Uncinaria stenocephala*. *A. caninum* is a dog-specific parasite, while *A*. *braziliense*, *A. ceylanicum*, and *U. stenocephala* affect both dogs and cats [1].

Hookworms cause zoonotic diseases and therefore have public-health significance [2]. The high frequency of occurrence of the larval stage of *A. braziliense* causes hookworm-associated cutaneous larva migrans (CLM). The larval stage of *A. caninum* is also implicated in CLM, which leads to common follicular dermatitis [2].

Hookworms are also of major veterinary importance. The adult stage of *A. caninum* and *A. ceylanicum* causes blood loss and anemia, which sometimes cause morbidity among young puppies and kittens [2].

The prevalence of *U. stenocephala* ranges from 0.3% to 33.3% in different regions, with high prevalence in urban, semirural, and rural populations in Spain (33.3%) and low prevalence in an urban dog population in Calgary, Canada (0.3%) [3,4]. Some reports have shown that hookworm prevalence rates are high among puppies and young adult dogs; on the other hand, some reports have shown higher prevalence rates among adult dogs [5].

In the present study, parasitological examination of fecal samples from 782 dogs were analyzed for the presence of *Uncinaria stenocephala*.

## Materials and Methods

### Ethical approval

The study protocol was designed according to the Ethics Committee of the Far Eastern Federal University.

### Study period and location

Between 1992 and 2014, fecal samples from 782 privately owned dogs of known age in Vladivostok, Russia, were examined for endoparasites. The reasons for obtaining these samples were routine examination, general health check, gastrointestinal disorders, and dermatitis. The prevalence dynamic in the study was not estimated due to an absence of data on parasite infection among dogs between 2000 and 2006 years.

### Sample collection and organization of data

Fresh fecal samples from these dogs were placed in individual plastic jars or packs with labels. Most of the samples were examined on the same day, while some samples were stored at 4-6°C for 3-4 days before examination. For diagnostic evaluation, data on clinical signs, dog breed, sex, and age were routinely provided (Table-1). Data on antiparasitic treatments were not included because unregulated and inadequate procedures for anthelminthic preventive therapy are frequently used by dog-owners. Replicate samples from the same dog were excluded from the data set. Based on age classifications, all the dogs were classified as puppies (<1 year of age), young adults (<2 years of age), and dogs older than 2 years.

**Table-1:** The prevalence of *Uncinaria stenocephala* in dogs.

Factor	Number investigated	Prevalence, %	Totally infected
Age			
Puppies	370	5.4	20
1-2 years of ages	205	8.3	17
Elder than 3 years of ages	163	4.3	7
Sex			
Male	416	7.2	30
Female	362	5.8	21
Season			
Summer	198	11.1	22
Autumn	243	7	17
Winter	167	2.4	4
Spring	174	4.6	8

### Fecal flotation method

The fecal examination was enabled through a standardized flotation method using a saturated salt solution containing NaNO_3_ (specific gravity [SG] 1.38) with a centrifugation step. An amount of 2-4 g of fecal material was placed in a 10 ml tube, suspended with NaNO_3_ solution and mixed thoroughly using a glass rod. The samples were stored in this salt solution for 20-30 min at room temperature. Helminth eggs and protozoan cysts that had come to the surface were then collected using a copper loop and placed on a slide, and some drops of glycerol were added.

It was found that the protozoan cysts could not be distinguished sufficiently through their morphological characteristics. Therefore, infections among the dogs caused by *Neospora caninum* and *Hammondia heydorni* were documented as *Sarcocystis oocysts*. A lack of morphological criteria for distinguishing these two protozoan species in terms of oocyst size or structure on microscopic examination had been noted previously [6].

### Statistical analysis

The data were analyzed using the Stata MP 14 software (StataCorp LLC, USA). Bivariate logistic regression was used to model the probability of infection in relation to a range of factors such as sex, gender, dog breed, and symptoms. Associations between predictor variables (independent variables) and infection (dependent variable) were determined in terms of odds ratios and p values and their 95% confidence intervals (Table-1).

## Results

Eggs of *U. stenocephala* eggs were found in fecal samples from 51 dogs (6.5%) aged between 1 month and 8 years. The highest prevalence rates were found among young adult dogs (8.3%), followed by puppies (5.4%); the lowest prevalence rates were found among dogs older than 3 years (4.3%). Analysis of the age distribution among *U. stenocephala*-positive dogs showed that in the puppy group, dogs aged 5-7 months were frequently affected by *U. stenocephala*. Among the dogs in the groups of young adults and adults older than 2 years, *U. stenocephala* was detected mainly in dogs aged 15-16 months and dogs aged 36 months, respectively (Table-1).

The prevalence was 5.8% among female dogs and 7.2% among male dogs. In general, the prevalence was higher among puppies of both sexes. Among *U. stenocephala*-positive young adult dogs, it was 50% in females and 42.3% in males. Female dogs older than 3 years of age were less frequently affected than males of this age (Table-1).

The affected dogs were most frequently registered in summer (June-August; 11.1%) and autumn (September-November; 7%). The number of infected dogs was lower in spring (April-May; 4.6%), and the least number of affected animals was found in winter (December-February; 2.4%) (Table-1).

The frequency of coinfections in *U. stenocephala*-positive dogs (n=51) was also analyzed. Coinfections with roundworms and protozoan parasites were frequently observed among the affected dogs (25.5%; n=13). In total, three types of coinfection were registered: Coinfection of *U. stenocephala* + *S. oocysts* was recorded in 19.1% of the dogs (n=10). This may have been related to higher prevalence rates for *S. oocysts* in the dogs (n=153; 19.5%). There were two cases of coinfection of *U. stenocephala* + *Toxocara canis* (3.9%), which may have been related to low prevalence of *T. canis* (7.4%). One case of coinfection of *Dipylidium caninum + U. stenocephala* (0.1%) also appeared. Other helminths, including *Toxascaris leonina, Diphyllobothrium latum*, *A. caninum*, *Taenia* spp., and flatworms from the Opisthorchiidae family, were also found in these dogs, but coinfections between *U. stenocephala* and one or more of these parasites were not found.

Bivariate logistic regression analyses were used to identify factors that might have contributed to increased risk of infection with *U. stenocephala*. These factors included host age, sex, season, and coinfections. These analyses showed that there was no positive relationship between *U. stenocephala* prevalence and sex. Puppies were less exposed to infection. There was a positive relationship between *Uncinaria* prevalence and the summer and autumn seasons. Absence of coinfection was identified as a risk factor for *U. stenocephala* infection (Table-2).

**Table-2:** Results of bivariate regression analysis.

Categories	OR	CI	p-value
Age			
Puppy	0.15	0.01-1.72	0.06
Young (13-36 months)	0.21	0.02-2.18	0.18
Adult (elder than 3 years)	0.11	0.01-1.11	0.12
Sex			
Female	0.76	0.38-1.51	0.43
Season			
Spring	0.68	0.56-0.19	0.55
Summer	0.22	0.01-0.07	0.01
Autumn	0.34	0.06-0.11	0.06
Coinfection			
*Coccidia*	1.81	1.19-7.56	0.02
*Toxocara canis*	1		
No	17.6	12.73-24.48	0.00

OR=Odds ratio, CI=Confidence interval

## Discussion

The fecal examination procedure was sufficient for making a clear diagnosis of parasite infection. For diagnosing *A. caninum* and *U. stenocephala* infection, fecal flotation is the most frequently used procedure. Flotation methods can be either qualitative or quantitative. Quantitative methods include the FLOTAC and McMaster methods and evaluate eggs per gram rate [7,8].

The flotation method used in the present study has good sensitivity for detecting most helminth species. NaNO_3_ solution has a higher SG (1.3) than zinc sulfate solution. It also has the same SG as sucrose (SD 1.36), which is also frequently used in standard procedures for coproscopic examination [9,10].

The hookworm *U. stenocephala* is a common intestinal parasite of domestic dogs in regions with a cold climate. The prevalence rates of *U. stenocephala* can vary in different regions, as a function of climate, living conditions, quality of veterinary care, and diagnostic resources [11,12]. In Russia, this species is not as frequently recorded in dogs as is *T. canis*. Data on parasite prevalence among dogs in Russia over the past 10 years show that *U. stenocephala* was recorded sporadically, mainly in the western part of Russia, with prevalence rates from 0.9 to 6.6% [13-15]. In our study, the prevalence rate was 6.6%, which was similar to the *U. stenocephala* prevalence rate recorded among dogs in Pyatigorsk [14].

Hookworm eggs and larvae are also sensitive to climatic conditions. *Ancylostoma caninum* larvae develop in warm soil shielded from direct sunlight, at temperatures of between 20 and 30°C. The larvae fail to develop at temperatures of <13°C and cannot survive at temperatures of <0°C and above 45°C [10]. *U. stenocephala* is less sensitive to climate conditions: Its larvae show different rates of development and ­longevity over the course of the year and can also survive at low temperatures.

In the present study, infected dogs were recorded in all seasons of the year, with peaks of infection in the summer and autumn. The high prevalence rates recorded in the summer and autumn were related to the herbage in paddocks, which can survive as sources of infection. The snow blanket that occurs in winter and early spring contributes toward reducing the risk of infection. In urban areas, environmental contamination by helminth eggs is a major source of infection. High levels of fecal contamination of local areas (in paddocks and parks) have also been correlated with higher infection rates [16].

Some general assumptions can be made regarding age differences in canine parasitic infection rates. Age-dependent prevalence rates can vary according to parasite species. For example, roundworms and hookworms are predominantly parasites of animals at the neonatal stage [17].

The association between age and parasite recurrence is due to the fact that the immature immune system of very young dogs is unable to generate long-term immunity against parasites over the course of these dogs’ development, while the immune system of older animals may begin to lose the anamnestic responses against previously seen parasites. Puppies are at higher risk of infection due to transmammary and transplacental transmission of some nematodes such as *T. canis* and *A. caninum* [18].

In the present study, *U. stenocephala* was found in dogs aged between 1 month and 8 years. Our results show that dogs of different ages can become infected with hookworms, in agreement with other reports [19,20]. However, adult dogs are less susceptible to *U. stenocephala* infection, due to anamnestic responses against previously seen parasites.

In this study, a higher prevalence was observed among male dogs than among female dogs. One possible explanation for the association between sex and parasite recurrence relates to the female host supremacy paradigm. This implies that female mammals are more resistant to parasitic infections than males because of gender-associated differences in exposure and the immunosuppressive properties of testosterone [21].

However, recent studies have shown that the role of the host’s immune system as the only effector of sex-associated differences in parasitism remains unclear and has been insufficiently explored. There is not uniformly implemented in the host-parasite pairs that have been studied most [21].

Multiple infections with different helminth species and with combinations of helminths plus protozoa are frequently observed in dogs. In the present study, two types of coinfection were found in the dogs. The most frequently observed coinfection was *U. stenocephala* + *S. oocysts*. This coinfection did not increase the duration of diarrhea or other gastrointestinal disorders. It was interesting that only one case of coinfection of *U. stenocephala + T. canis* was found. The source of infection regarding both of these species is contamination of the environment with eggs and larvae. Low levels of coinfection of *U. stenocephala* + *T. canis* relate to low prevalence rate of *T. canis*. Dogs living in different districts may live in paddocks with different characteristics and varying contamination rates. No other types of coinfection were recorded, given the low prevalence rates of helminth species, differences in their life cycles and different sources of infections.

## Conclusion

The present study showed that male dogs and young dogs were most susceptible to *U. stenocephala* infection.

## Authors’ Contributions

All authors designed, planned, drafted, and revised the manuscript. AVT contributed to statistical analysis. IVG, PVF, and TVT contributed to fecal examination. MYS and EMK contributed to text preparation and edition. All authors read and approved the final manuscript.
